# Transgenic plants expressing ω-ACTX-Hv1a and snowdrop lectin (GNA) fusion protein show enhanced resistance to aphids

**DOI:** 10.3389/fpls.2014.00673

**Published:** 2014-11-28

**Authors:** Erich Y. T. Nakasu, Martin G. Edwards, Elaine Fitches, John A. Gatehouse, Angharad M. R. Gatehouse

**Affiliations:** ^1^Plant-Insect Molecular Interactions Group, Newcastle Institute for Sustainability, School of Biology, Newcastle UniversityNewcastle upon Tyne, UK; ^2^Capes Foundation, Ministry of Education of BrazilBrasília, Brazil; ^3^School of Biological and Biomedical Sciences, Durham UniversityDurham, UK

**Keywords:** insect-resistant transgenic plants, *Myzus persicae*, *Arabidopsis*, Hv1a/GNA, fusion proteins, *Sitobion avenae*

## Abstract

Recombinant fusion proteins containing arthropod toxins have been developed as a new class of biopesticides. The recombinant fusion protein Hv1a/GNA, containing the spider venom toxin ω-ACTX-Hv1a linked to snowdrop lectin (GNA) was shown to reduce survival of the peach-potato aphid *Myzus persicae* when delivered in artificial diet, with survival <10% after 8 days exposure to fusion protein at 1 mg/ml. Although the fusion protein was rapidly degraded by proteases in the insect, Hv1a/GNA oral toxicity to *M. persicae* was significantly greater than GNA alone. A construct encoding the fusion protein, including the GNA leader sequence, under control of the constitutive CaMV 35S promoter was transformed into *Arabidopsis*; the resulting plants contained intact fusion protein in leaf tissues at an estimated level of 25.6 ± 4.1 ng/mg FW. Transgenic *Arabidopsis* expressing Hv1a/GNA induced up to 40% mortality of *M. persicae* after 7 days exposure in detached leaf bioassays, demonstrating that transgenic plants can deliver fusion proteins to aphids. Grain aphids (*Sitobion avenae*) were more susceptible than *M. persicae* to the Hv1a/GNA fusion protein in artificial diet bioassays (LC_50_ = 0.73 mg/ml after 2 days against LC_50_ = 1.81 mg/ml for *M. persicae*), as they were not able to hydrolyze the fusion protein as readily as *M. persicae*. Expression of this fusion protein in suitable host plants for the grain aphid is likely to confer higher levels of resistance than that shown with the *M. persicae*/*Arabidopsis* model system.

## INTRODUCTION

Aphids significantly impact agricultural and horticultural crops, either by causing direct damage to plants through feeding on the phloem, or indirectly by acting as vectors for plant pathogenic viruses. Aphid control relies heavily on the use of synthetic insecticides. Intensive pesticide use has positively selected aphid genotypes that are resistant to carbamates and organophosphates, which inhibit the enzyme acetylcholinesterase, and pyrethroids, which target sodium channels (Devonshire et al., 1998). More recently, aphid resistance to neonicotinoids, nicotinic acetylcholine receptor (nAChR) agonists, has also been reported (e.g., Puinean et al., 2010). Therefore, alternatives for chemical control and the development of insecticides with different modes of action are needed.

Spider venom neurotoxins offer a high degree of biological activity, providing an attractive source for novel pest management strategies (King, 2007). However, there are major drawbacks to the use of these peptides, particularly as topical sprays, as they are unlikely to be rapidly absorbed through the insect cuticle to reach their site of action and are prone to degradation in the environment (Fitches et al., 2004a). Should they survive the application process and be taken up by the insect, they are then unlikely to survive the conditions of the insect gut (Fitches et al., 2004a) or be delivered across the midgut epithelium to the correct targets within the insect (Tedford et al., 2004). The discovery that snowdrop lectin *Galanthus nivalis* agglutinin (GNA) remains stable and active within the insect gut after ingestion, and that it is able to cross the midgut epithelium (Powell et al., 1998), provided an opportunity for its use as a ‘carrier molecule’ to deliver other peptides to the circulatory system of target insect species (Fitches et al., 2002).

The venom peptide ω-ACTX-Hv1a (Hv1a) from the Australian funnel web spider *Hadronyche versuta* (Rainbow) acts as a calcium channel blocker in the insect central nervous system (CNS; Bloomquist, 2003). It has proven to be lethal to a broad range of insects (Atkinson et al., 1998), but causes no inhibition to mammalian voltage-gated calcium channel currents (Fletcher et al., 1997). However, the peptide does not show oral toxicity to insects (Tedford et al., 2004). Fitches et al. (2012) fused it to the carrier molecule GNA. The authors were able to demonstrate effective delivery of the peptide to *Mamestra brassicae* hemolymph when ingested and that it reached the Hv1a site of action in the central nerve cord. Furthermore, the neurotoxin portion of the Hv1a/GNA fusion protein was modified with an amino acid substitution (K34Q) in order to improve its stability during yeast expression (Pyati et al., 2014).

The peach-potato aphid, *Myzus persicae*, is a cosmopolitan, generalist species that feeds on more than thirty different plant families, including commercially important crops, being capable of transmitting more than 100 viral diseases (van Emden et al., 1969). The grain aphid *Sitobioin avenae* is a semi-specialist species that infests plants from the Poaceae family, being an important pest of wheat (*Triticum aestivum*) in China (Wang et al., 2011) and Western Europe (Larsson, 2005). The present study demonstrates that the fusion protein Hv1a/GNA is toxic toward both the peach-potato aphid and the grain aphid. Furthermore, transgenic *Arabidopsis* plants expressing the fusion protein were effective at controlling *M. persicae,* thus demonstrating the potential of using fusion protein technology for aphid control.

## MATERIALS AND METHODS

### PROTEIN EXPRESSION AND PURIFICATION

*Pichia pastoris* (SMD1168H strain) was transformed with genes encoding GNA (Raemaekers et al., 1999) or Hv1a/GNA (Pyati et al., 2014) and fermentation carried out in a Bio Console ADI 1025 (Applikon) fermenter (2 l vessels), with a continuous 50% glycerol feed. After expression, cultures were centrifuged at 7500 *g* for 30 min and the supernatant collected. Recombinant GNA was purified by hydrophobic interaction chromatography on a phenyl-sepharose resin packed into a Pharmacia XK16 column. Fractions containing GNA were reloaded onto a size-exclusion column (HiPrep^TM^ 16/60 Sephacryl S-100, GE-Healthcare). Following purification, recombinant proteins were dialyzed, freeze-dried and stored at -20°C. For His-tagged Hv1a/GNA purification, supernatants were diluted in binding buffer (0.02 M sodium phosphate, 0.4 M NaCl, pH 7.4). Samples were loaded onto a HisTrap^TM^ (GE Healthcare) column and then eluted with binding buffer containing 0.2 M imidazole. After purification, samples were extensively dialyzed in water and freeze-dried. The concentration of Hv1a/GNA was estimated by comparing band intensities with known amounts of GNA on SDS-PAGE, as described Down et al. (2006).

### ARTIFICIAL DIET BIOASSAYS

*Myzus persicae* were kept on Chinese cabbage plants (*Brassica rapa*) at 25°C, 16:8 (L:D), whereas *S. avenae* were reared on wheat (*T. aestivum*), at 20°C, 16:8 (L:D). Prior to bioassays, apterous adult aphids were transferred from plants to 90 mm diameter Petri dishes containing artificial diet (Febvay et al., 1988) in Parafilm sachets as described by Down et al. (1996), and allowed to reproduce for 24 h. Neonate aphids (ten per Petri dish) were collected and exposed to one of the four treatments in artificial diet: (i) artificial diet alone (negative control), (ii) 1 mg/ml GNA, (iii) 0.5 mg/ml Hv1a/GNA, or (iv) 1 mg/ml Hv1a/GNA. Mortality was recorded daily for 8 days and diets were changed every 48 h. Thirty aphids per treatment (in three Petri dishes) were used for *S. avenae* bioassays, and 70 aphids/treatment (in seven Petri dishes) were used for *M. persicae* bioassays.

Fecundity of *M. persicae* was evaluated by continuously feeding neonate aphids with Hv1a/GNA or GNA at 0.25 mg/ml for 9 days, as aphids do not reach adulthood when fed higher concentrations of fusion protein. Three cages containing 10 aphids were used for each treatment, and the cumulative number of nymphs produced/day/adult was recorded. For evaluating the effects of GNA or Hv1a/GNA on *M. persicae* development, three replicates of ten 2-days old aphids were given artificial diet alone, GNA, or Hv1a/GNA at 1 mg/ml of artificial diet. Aphid lengths (from head to cauda) were measured on the first 3 days by using a graticule. For all bioassays, environmental conditions were as stated above for rearing.

### UPTAKE OF Hv1a/GNA BY APHIDS

Neonate *M. persicae* and *S. avenae* were fed for 24 h on artificial diet containing Hv1a/GNA at 0.5 or 1 mg/ml. Insects (10–15) were either collected, flash frozen in liquid nitrogen and macerated in SDS sample buffer for protein extraction, or transferred to Petri dishes containing artificial diet without added proteins for a pulse-chase experiment. After 24 h, those aphids were collected and their proteins extracted as described above. Honeydew from each treatment was collected from the bottom of the Petri dishes, 24 h after the beginning of the assays. Samples were heat-denatured and separated in 15% SDS-PAGE. Proteins were then transferred to nitrocellulose membranes and the uptake of fusion proteins evaluated by western blot using anti-GNA antibodies (1:5000 dilution) and enhanced luminol-based chemiluminescent (ECL) substrate, as previously described Fitches et al. (2012).

### PLANT TRANSFORMATION

**FIGURE 1:**
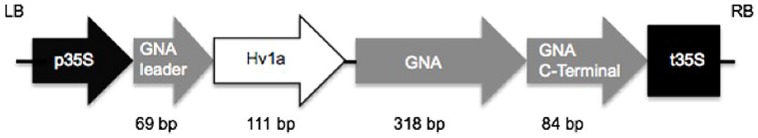
**Structure of plant constructs in pK2GW7 vector**.

A sequence coding for Hv1a/GNA was synthesized with *Arabidopsis thaliana* codon usage for optimal plant expression (ShineGene Molecular Biotech, Inc., Supplementary material). Primers containing attB1 and attB2 sites (**Table 1**) were used to amplify the gene via PCR (30 cycles of 98°C for 10 s, 55°C for 30 s, and 72°C for 30 s, with a final extension step of 7 min), which was then transferred to pDONR vectors using BP clonase reaction (Gateway^®^, invitrogen^TM^). The construct (**Figure 1**) was composed of a GNA precursor leader sequence (van Damme et al., 1991), followed by the venom toxin Hv1a (K34Q; Pyati et al., 2014), a linker region composed of three alanines, and GNA followed by its C-terminal extension (van Damme et al., 1991). The GNA precursor leader and the C-terminal extension sequences were added to the construct in order to provide correct folding and trafficking of the fusion protein to the phloem sap (Rao et al., 1998). Constructs were electroporated into *Escherichia coli* Top10 and plasmids extracted from positive colonies. In a subsequent step, the gene coding for the fusion protein was transferred from the pDONR to pK2GW7 vector (Karimi et al., 2002) via LR clonase using Gateway^®^ technology (Invitrogen^TM^).

**Table 1:** Primers used to add *att*B sites (in bold) to Hv1a/GNA coding sequence.

Primer	Sequence
Sense	5′**GGGGACAAGTTTGTACAAAAAAGCAGGCT**AT GGCTAAGGCAAGTCTCCT3′
Antisense	5′**GGGGACCACTTTGTACAAGAAAGCTGGGT**TTACTT TGCCGTCACAAGC3′

Expression constructs were finally electroporated into *Agrobacterium tumefaciens* C58C1, and antibiotic resistance was used to screen transformed colonies. *Arabidopsis thaliana* (var. Columbia) were transformed with *A. tumefaciens* following the floral dip method described by Clough and Bent (1998). Seeds were harvested, surface-sterilized and spread on plates with Murashige-Skoog medium containing 50 μg/mL kanamycin. Plates were kept at 4°C for 48 h in order to break seed dormancy and then transferred to environmentally controlled growth rooms (16:8 h L:D, 22°C day and 17°C night). Putative transformed plantlets were transferred to plastic pots containing soil (John Innes No. 2). Transformation was confirmed via PCR using the same conditions described above and by western blots. Protein expression was estimated by macerating a known amount of leaf tissue in 1.5x SDS loading buffer containing 2-mercaptoethanol (1 mg/10 μl). Samples were macerated, boiled for 5 min and centrifuged at 13,000 *g* for 2 min. Supernatants (20 μl) and GNA standards (25, 50, and 100 ng), used to estimate Hv1a/GNA concentrations, were loaded onto 15% SDS-PAGE. After electrophoresis, proteins were transferred to nitrocellulose membranes. Fusion proteins and GNA standards were probed with anti-GNA antibody as described above.

### BIOASSAYS WITH TRANSGENIC PLANTS

Transgenic F_3_ *Arabidopsis* plants homozygous for the gene expressing Hv1a/GNA were used in bioassays with *M. persicae* only, as *S. avenae* does not feed on crucifers. Leaves from two homozygous transgenic lines (1.2a and 1.3b) and non-transgenic *Arabidopsis* (negative control) were detached from approximately five-week-old plantlets (ca. 30 plants/line were used, ensuring that leaves taken were of comparable age). Their petioles were immersed in 0.5% agar contained in 1.5 ml plastic tubes, which were then individually placed in 450 ml plastic boxes. Six replicates of five aphids were used for each treatment. Aphids were kept at 25°C, 16:8 (L:D). Leaves were replaced every 2 days and the number of alive aphids recorded daily for 6 days. Survival analysis was carried out as described below (see Statistical Analyses).

### STATISTICAL ANALYSES

Log-rank Kaplan–Meier survival analyses with pairwise comparisons were carried out using Sigmaplot 11 (2008).

Data recorded for length and fecundity of *M. persicae* exposed to GNA or Hv1a/GNA via artificial diet (see Artificial Diet Bioassays) were evaluated by one-way ANOVA. *Post hoc* pairwise multi-comparisons were carried out using Holm–Sidak method. The median lethal concentrations (LC_50_) of Hv1a/GNA against *M. persicae* and *S. avenae* were calculated by plotting log dose (0, 0.5, 1, and 2 mg/ml) vs. probit of corrected mortalities (Abbott, 1925; Miller and Tainter, 1944; Randhawa, 2009).

## RESULTS

### DEMONSTRATION OF INSECTICIDAL ACTIVITY OF Hv1a/GNA AGAINST THE PEACH-POTATO APHID *M. persicae*

**FIGURE 2:**
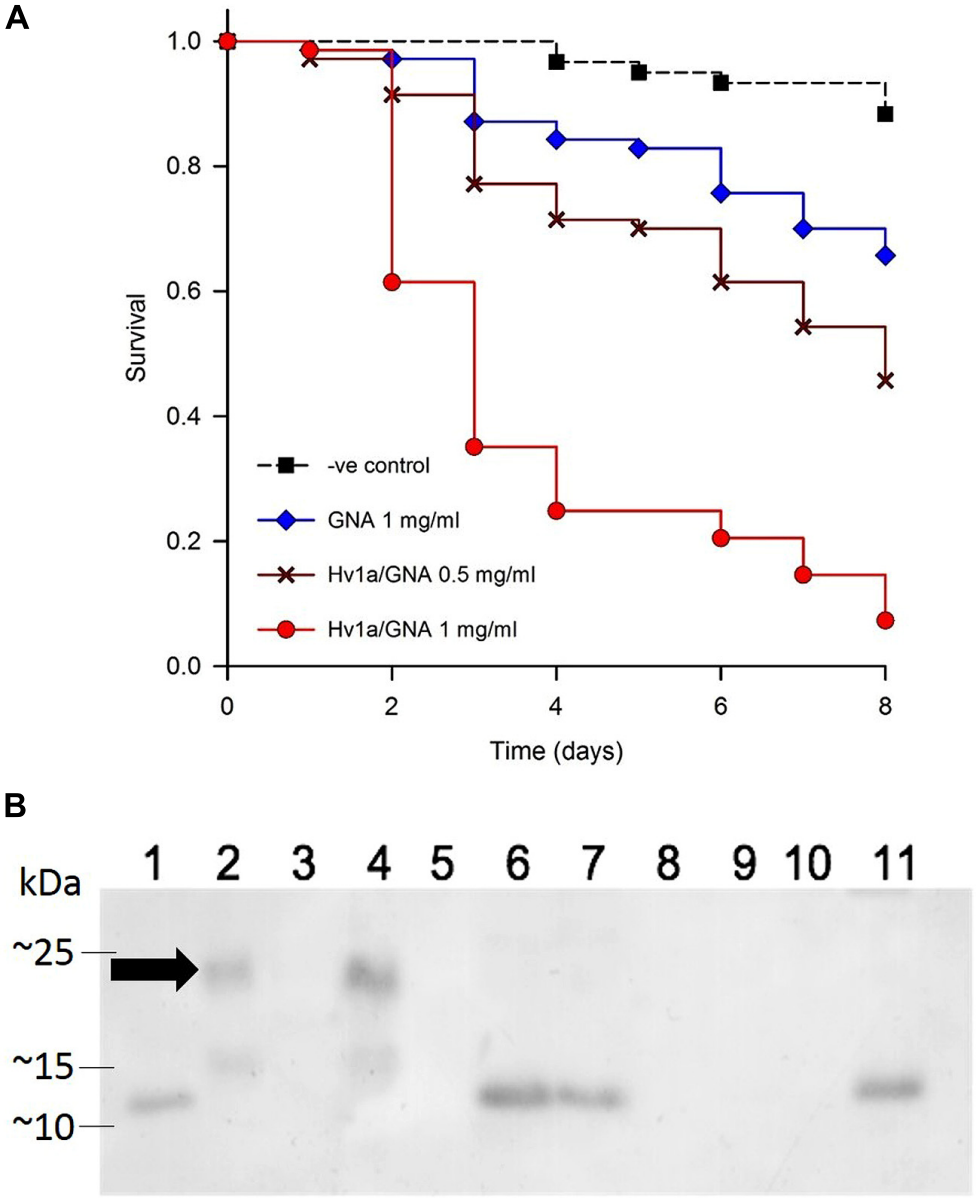
**Effects of Hv1a/GNA on *M. persicae* via artificial diet. (A)** Survival analysis of *M. persicae* fed on artificial diet alone or containing either GNA or Hv1a/GNA (*n* = 70 aphids/treatment). **(B)** Western blot analysis of different samples taken from *M. persicae* fed with Hv1a/GNA. (1) GNA 100 ng; (2) Hv1a/GNA 100 ng; (3) Aphid diet (negative control); (4) Aphid diet + Hv1a/GNA (0.5 mg/ml); (5) Adult aphids (negative control); (6) Adult aphids after feeding for 24 h with Hv1a/GNA; (7) Adult aphids chase experiment; (8) Aphid nymphs (negative control); (9) Aphid nymphs from adults fed with Hv1a/GNA; (10) Honeydew (negative control); (11) Honeydew from aphids feeding on 0.5 mg/ml Hv1a/GNA. Arrow shows migrating pattern of intact Hv1a/GNA.

The toxicity of Hv1a/GNA was assayed using neonate (<24 h) *M. persicae* nymphs fed recombinant fusion protein Hv1a/GNA at 0.5 or 1 mg/ml of artificial diet. Results are shown in **Figure 2**. Aphids presented increased mortality on fusion protein treatments from the second and third days after the start of experiments. Survival curves differed from each other (*p*< 0.001), and pairwise multiple comparisons showed significant differences between all treatments (*p* < 0.05). Hv1a/GNA showed higher levels of toxicity toward *M. persicae* than that of GNA alone, demonstrating its increased toxicity against this species. When fed at a concentration of 1 mg/ml, the fusion protein Hv1a/GNA resulted in more than 90% decrease in survival after 8 days, whereas GNA alone at 1 mg/ml resulted in less than 35% reduction (**Figure 2A**). Subsequently, a dose/response assay was carried out using five different protein concentrations of either GNA or Hv1a/GNA. When continuously feeding on diets with test proteins, aphids were once more shown to be significantly more susceptible to the fusion protein than to GNA (data not shown, *p* < 0.05), with an estimated LC_50_ for the fusion protein of 1.81 mg/ml after 2 days. It was not possible to reliably calculate the LC_50_ for GNA alone with the concentrations used, as 50% mortality was not achieved at the doses fed, and mortalities did not always increase linearly with increased concentrations of the lectin.

Immunoassays by western blot analysis of aphids fed on artificial diet containing Hv1a/GNA demonstrated that fusion proteins were rapidly digested by *M. persicae.* Anti-GNA antibodies recognized a single band of around 10 kDa (**Figure 2B**) in extracts from whole aphids fed with Hv1a/GNA in a pulse-chase experiment, 24 h after exposure. Furthermore, the ∼10 kDa band was also detected in the honeydew, suggesting that the fusion protein is cleaved in the gut, and no evidence of intact Hv1a/GNA was observed. Although GNA and fusion proteins are internalized by homopterans (Down et al., 2006), it was not transmitted to nymphs descended from aphids feeding on Hv1a/GNA.

### EFFECTS OF Hv1a/GNA ON DEVELOPMENT AND FECUNDITY OF *M. persicae*

**FIGURE 3:**
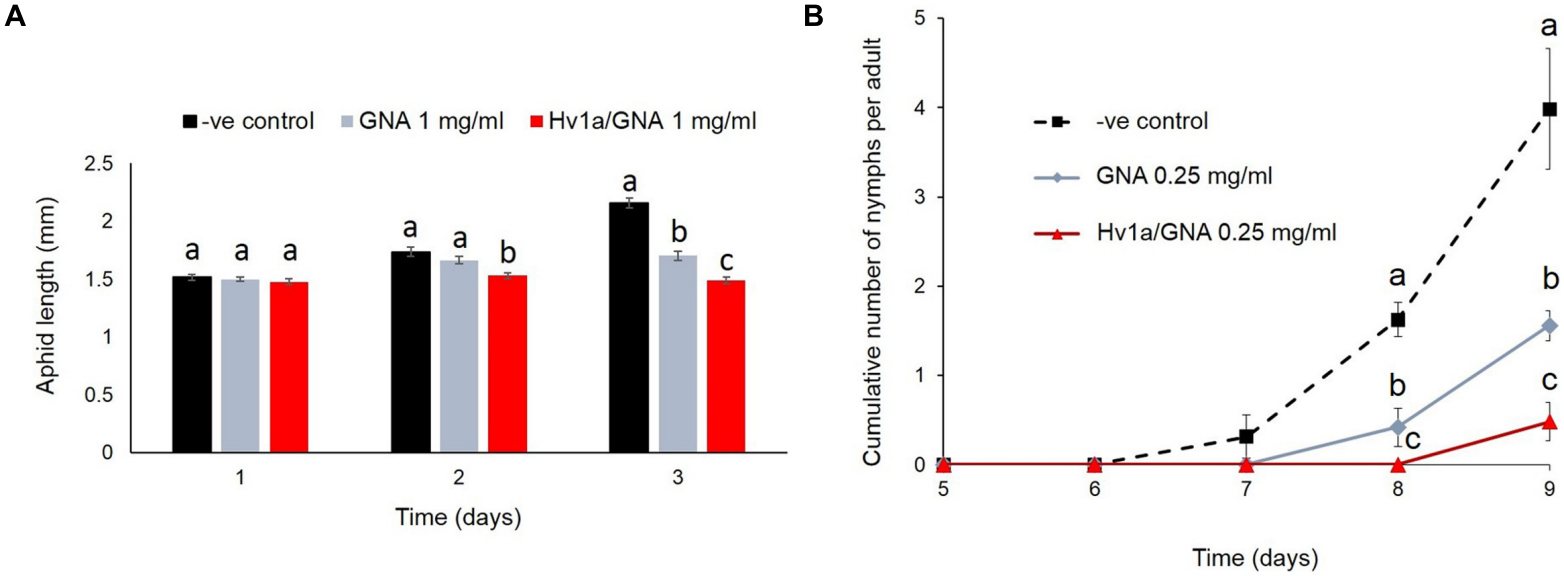
**Effects of Hv1a/GNA and GNA on *M. persicae* development and fecundity. (A)** Aphid length after feeding on either GNA or Hv1a/GNA at 1 mg/ml of artificial diet (*n* = 30 aphids/treatment). **(B)** Cumulative number of nymphs/adult produced by aphids fed with either GNA or Hv1a/GNA. For both graphs, different letters represent significant difference between treatments (*p* < 0.05); bars represent means ± SEM.

Myzus *persicae* nymphs were significantly smaller than controls (*p* < 0.001) following 2 days continuously feeding on diet containing Hv1a/GNA at 1 mg/ml, although they presented similar sizes at the beginning of the experiments (*p* = 0.98). After 3 days, insects fed on 1 mg/ml Hv1a/GNA or GNA were approximately 30 and 20% smaller than controls, respectively (*p* < 0.001; **Figure 3A**). Additionally, when compared to controls, the cumulative number of nymphs produced per adult was significantly reduced on aphids fed with GNA (ca. 69%, *p*= 0.002) or Hv1a/GNA at 0.25 mg/ml (>90% reduction, *p* < 0.001) after 9 days from the start of the experiment (**Figure 3B**). It was not possible to test the effects of this recombinant protein at a higher dose of 1 mg/ml, as no nymphs reached adulthood (**Figure 2**).

### EFFECTS OF Hv1a/GNA FUSION PROTEIN ON *Sitobion avenae* SURVIVAL

**FIGURE 4:**
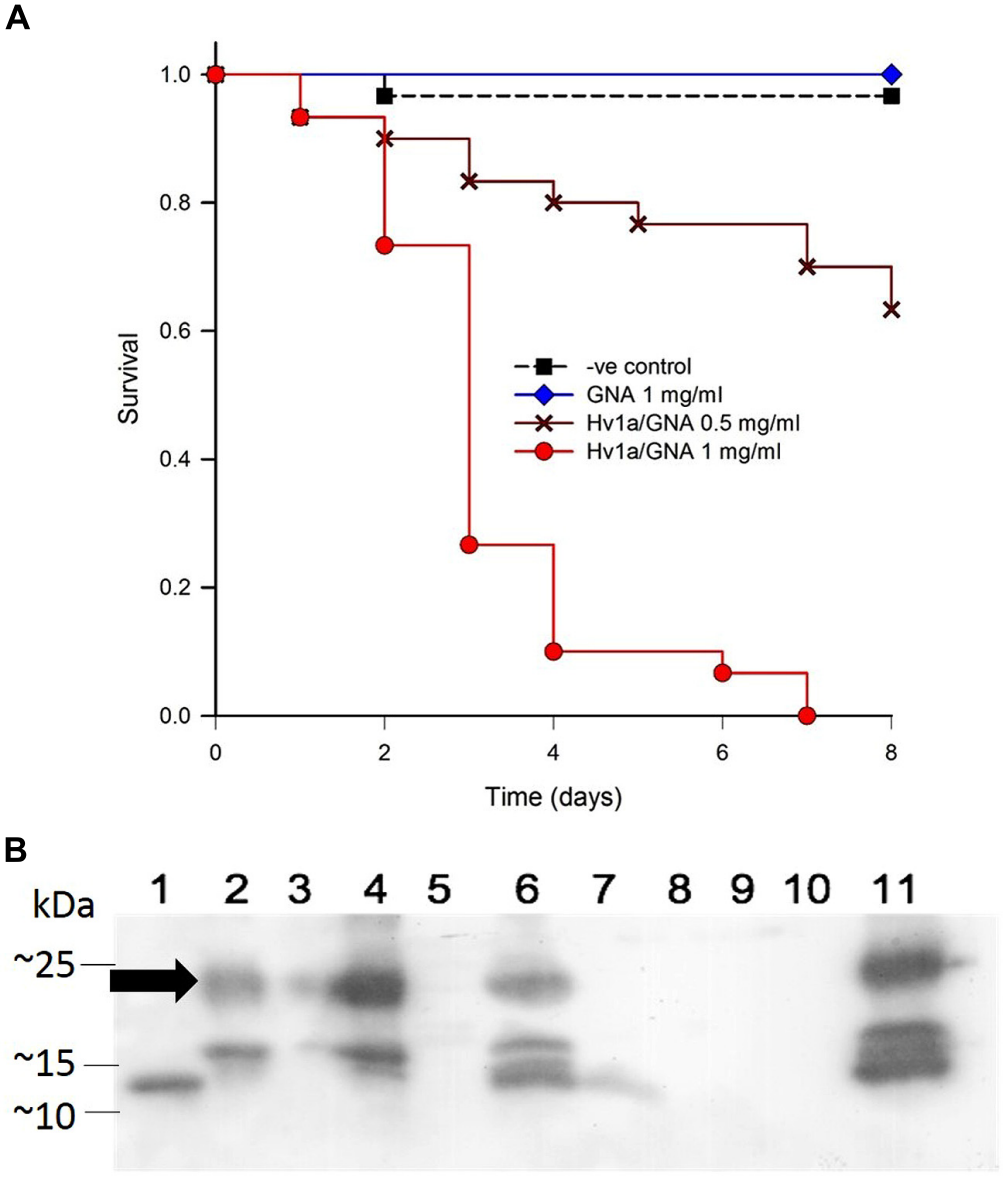
**Biological activity of Hv1a/GNA against *Sitobion avenae.* (A)** Survival analysis of aphids fed with artificial diet alone or diet containing either GNA or Hv1a/GNA (*n* = 30 aphids/treatment). **(B)** Western blot analysis demonstrating the fate of Hv1a/GNA following ingestion by *S. avenae*. (1) GNA 100 ng; (2) Hv1a/GNA 100 ng; (3) Aphid diet (negative control); (4) Hv1a/GNA at 0.5 mg/ml of artificial diet; (5) Adult aphids (negative control); (6) Adult aphids after feeding for 24 h with Hv1a/GNA; (7) Adult aphids chase experiment; (8) Aphid nymphs (negative control); (9) Aphid nymphs from adults fed with Hv1a/GNA; (10) Honeydew (negative control); (11) Honeydew from aphids feeding on 0.5 mg/ml Hv1a/GNA. Arrow indicates position of intact Hv1a/GNA.

A bioassay with a semi-specialist aphid species, the grain aphid *Sitobium avenae*, was carried out to test the efficacy of Hv1a/GNA against this important pest when fed in liquid diet. Following Kaplan–Meier Survival analysis, significant differences between survival curves were found with bioassays using *S. avenae* (*p* < 0.001). Pairwise multiple comparisons (Holm-Sidak) revealed non-significant differences between GNA and control treatments (*p* = 0.317), while Hv1a/GNA at 0.5 and 0.1 mg/ml differed from all other treatments (*p* < 0.05; **Figure 4A**). These results demonstrate that *S. avenae* is more susceptible to Hv1a/GNA than *M. persicae*, and while GNA alone did not significantly affect survival, the fusion protein rapidly induced mortality, with LC_50_ of 0.73 mg/ml after 2 days, in contrast with a 2.4-fold higher LC_50_ for *M. persicae* (1.81 mg/ml).

Western blot analyses show that the grain aphid, as opposed to *M. persicae*, does not readily cleave the fusion protein. Intact Hv1a/GNA was detected in whole grain aphids feeding on fusion protein and also in their honeydew. Limited proteolysis is suggested by the appearance of additional immunoreactive bands of lower molecular mass than that of intact fusion protein in Hv1a/GNA fed aphid and honeydew samples. Only after 24 h, as shown in the chase experiment, is the fusion protein completely cleaved (**Figure 4B**).

### EXPRESSION OF Hv1a/GNA IN *Arabidopsis*

**FIGURE 5:**
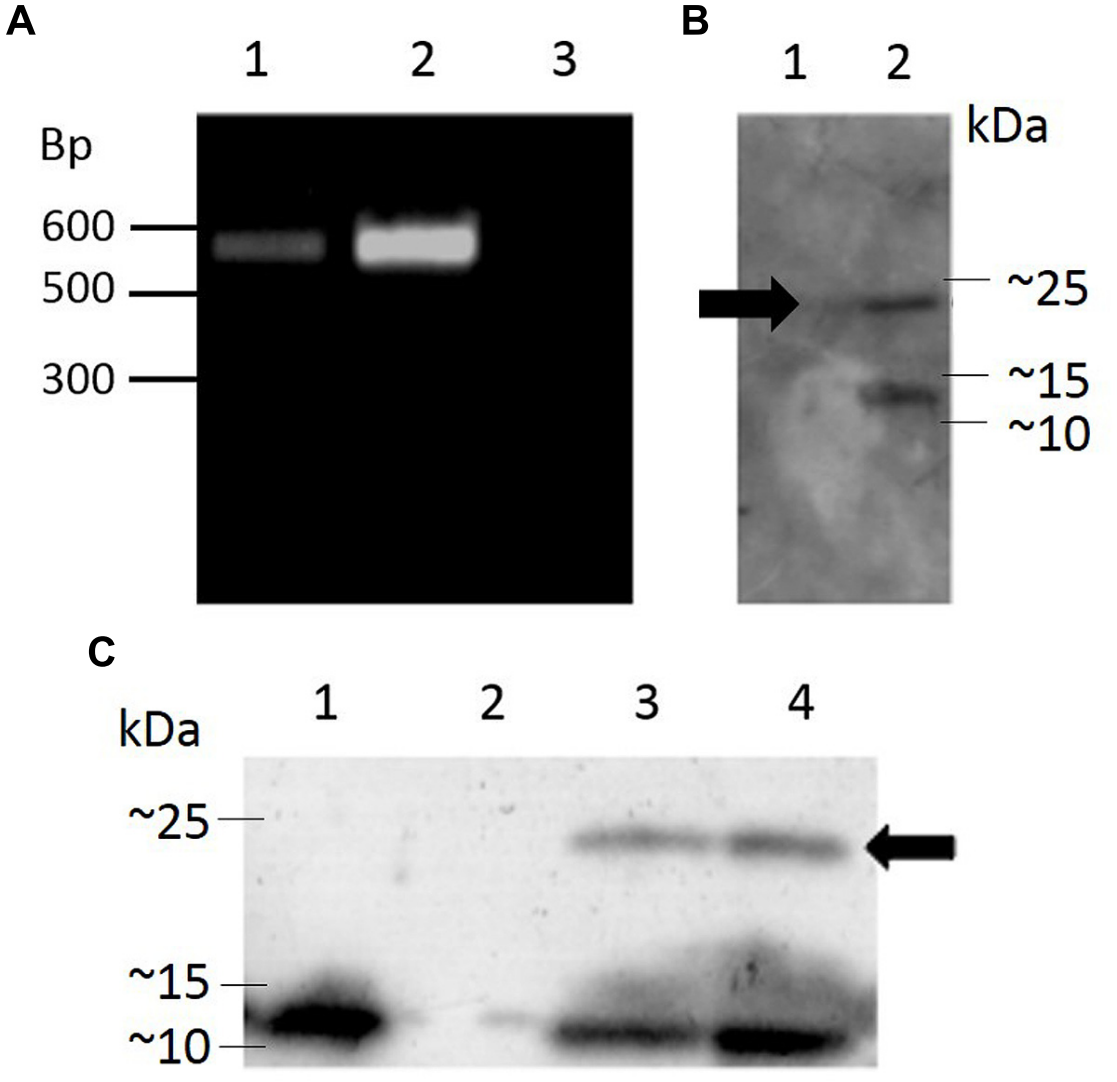
**Genomic integration of a coding sequence for Hv1a/GNA in *Arabidopsis* and expression analysis. (A)** PCR-positive plants; lanes 1 and 2, transformed plants, lane 3, untransformed plant (negative control). **(B)** Western blot showing expression of Hv1a/GNA (position shown by arrow) in F_0_ plants; lane 1, negative control (untransformed plant), lane 2, PCR-positive plant. **(C)** Expression of Hv1a/GNA (arrow) in homozygous plants. Lane 1, positive control (100 ng GNA), lane 2, negative control (untransformed plant), lanes 3 and 4, two different homozygous events.

Transgenic *Arabidopsis thaliana* plants harboring the pK2GW7 vector carrying the sequence for Hv1a/GNA under the control of the CaMV 35S promoter were generated using the *Agrobacterium tumefaciens*-mediated floral dip technique. After selection of T_0_ seeds on plates containing kanamycin, a transformation efficiency of 2.67 ± 0.46% (average number of kanamycin-resistant seeds ± SEM) was obtained from seven independent events. Integration of the transgene cassette was investigated by PCR (**Figure 5A**) and positive plants were self-pollinated in order to generate homozygous lines for the Hv1a/GNA fusion protein.

Western blot of leaf extracts from plants carrying Hv1a/GNA gene demonstrate that the fusion protein was expressed in T_0_ and homozygous F_3_ plants (**Figures 5B,C**, respectively). The ∼25 kDa band corresponding to the intact fusion protein is detected along with another lower molecular weight protein that also reacts with anti-GNA antibody. The lower molecular weight cleavage product was also present when the fusion protein is expressed in *P. pastoris*. This result indicates that the plant cleaves Hv1a/GNA following translation, and further improvements and alterations to the peptide structure would benefit its expression in heterologous systems. Quantification of expression was carried out by comparing intensity of Hv1a/GNA bands from known amounts of leaf extracts compared to GNA standards in western blots. It was estimated that the fusion protein was being expressed at 25.6 ± 4.1 ng/mg fresh weight (F.W.) leaf tissue.

### PERFORMANCE OF *M. persicae* IN PLANTA: DETACHED LEAVES BIOASSAY

**FIGURE 6:**
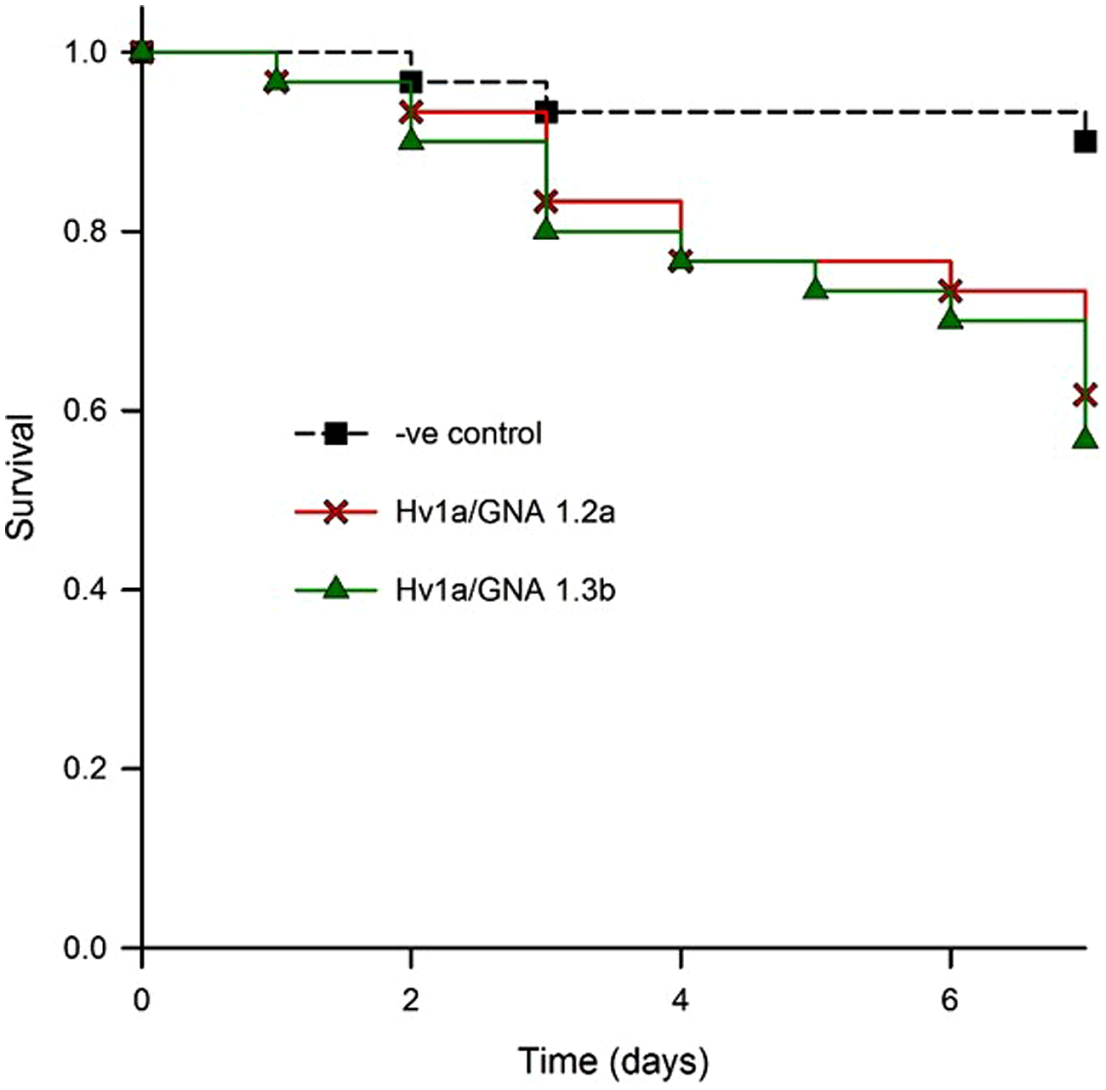
**Evaluation of biological activity of Hv1a/GNA expressed in *Arabidopsis* leaves.** Kaplan–Meier survival analysis of *M. persicae* on detached leaves of two different homozygous lines compared with non-transformed controls (negative control).

In order to test the efficacy of fusion proteins expressed in plants against aphids, a bioassay with transgenic *Arabidopsis* was set. Two homozygous lines (designated 1.2a and 1.3b) from independently transformed plants were assayed for aphid resistance. Leaves were detached from plants and their petioles immersed in 0.5% agar. When compared to non-transformed controls, aphids feeding on both events showed similar survival patterns, with significantly increased levels of aphid mortality (K-M, *p* = 0.014; control vs. 1.2a, *p*= 0.01; control vs. 1.3b, *p*= 0.003; 1.2a vs. 1.3b, *p* = 0.691); aphid survival was reduced to around 60% after 7 days (**Figure 6**). The corrected mortality using Abbott’s formula for 1.2a was 29.6 and 37% for 1.3b.

## DISCUSSION

Aphids are important crop pests that are difficult to control, as they possess high rates of reproduction and some species feed on plant parts that are inaccessible to insecticide applications. Therefore, transgenic plants expressing genes conferring aphid resistance would be valuable tools for managing their populations. To this end, different strategies, including expression of lectins (Down et al., 1996; Chang et al., 2003), proteinase inhibitors (Rahbé et al., 2003; Carrillo et al., 2011; Zhang et al., 2012) and alarm pheromones (Beale et al., 2006) have been investigated. These approaches commonly result in plants presenting modest effects on aphid survival, having greater outcomes on fitness parameters, such as size and fecundity, or behavior.

The fusion protein presented significant levels of toxicity when compared to GNA alone in artificial diet bioassays. Sub-lethal effects of Hv1a/GNA on aphid size and fecundity, which have previously been reported for GNA (Down et al., 1996; Sauvion et al., 1996), were also recorded in the present study. Toxicity of GNA to the peach-potato aphid has been previously assayed (Sauvion et al., 1996), and transgenic plants expressing this particular lectin generally offer low levels of insect control (Hilder et al., 1995; Down et al., 1996; Stoger et al., 1999). The other component of the fusion protein, Hv1a, is highly toxic toward *M. persicae* when injected into the hemocoel, but innocuous when ingested (Pal et al., 2013). The high levels of toxicity of the fusion protein obtained in the present study following ingestion can be attributed to the transport of the intact and functionally active Hv1a peptide to its sites of action within the insect’s body by the GNA carrier.

Even though other fusion proteins encompassing GNA as the carrier molecule have been tested against homopterans via artificial diet (e.g., Trung et al., 2006 tested ButaIT/GNA against *Nilaparvata lugens*; Down et al., 2006 tested SFI1/GNA against *M. persicae* and *N. lugens*), this is the first time a representative of these biopesticides is delivered to insects via transgenic plants. *Myzus persicae* was targeted not only because of its status as a pest for several crop species, but also because it feeds on *Arabidopsis* plants, thus providing a valuable proof of concept of expressing GNA-based fusion proteins for insect control. Regarded as a generalist, this aphid can infest several plant species, being able to cope with different diet regimes. Consequently, the observed increased proteolytic activity when compared to *S. avenae* might play an important role for the resilience of this species and its extended host range. In the present study, Hv1a/GNA was readily cleaved by *M. persicae* gut proteases, as demonstrated by western blots of honeydew material. It has been previously demonstrated that proteolysis can significantly impact the effectiveness of fusion proteins (Fitches et al., 2004b), as the venom peptide on its own, without a carrier molecule, is not transported to its sites of action within the insect’s body. However, as aphids fed continuously on diets and plants containing Hv1a/GNA, minute amounts of indigested fusion protein would have crossed the gut, reaching Hv1a sites of action in the CNS. This can be ascertained by two observations. Firstly, the magnified toxicity of the fusion protein was markedly higher than GNA alone. Secondly, expression of GNA in transgenic potatoes can affect fecundity, but not survival of *M. persicae* (Gatehouse et al., 1996) and *Aulacorthum solani* (Down et al., 1996). In this work, although transgenic plants caused aphid mortality, expression levels were still insufficient to significantly influence reproduction. Stoger et al. (1999) report that expression of GNA in wheat plants only affects *S. avenae* fecundity at expression levels greater than 0.04% total soluble protein. A more efficient expression system would therefore benefit aphid control using fusion proteins. Additionally, Hv1a/GNA is partially cleaved when expressed in *Arabidopsis*, resulting in a product of the same size as GNA (**Figures 5B,C**), indicating that the triple alanine linker between the spider venom toxin and GNA is a potential cleavage site.

As aphids feed on the phloem sap, the use of a phloem-specific promoter would be desirable, avoiding unnecessary expression and reducing the chances of non-target organisms from being exposed to the fusion protein; however, expression in chloroplasts proved to be effective in delivering *Pinellia ternate* agglutinin to *M. persicae*, reducing its growth rate by up to 90% (Jin et al., 2012). Previous work has shown that GNA expression in wheat under constitutive promoters was considerably higher than when using phloem-specific promoters, and the control of *S. avenae* comparatively more efficient (Stoger et al., 1999). Similarly, Rao et al. (1998) report that GNA expressed under either the phloem-specific promoter *RSs1* (from the rice sucrose synthase gene) or the constitutive promoter *ubi1* (from the maize ubiquitin gene) showed equivalent insecticidal effects toward the sap-sucking homopteran *Nilaparvata lugens.* This study also showed that the GNA molecule was present in the phloem sap in both cases, as a consequence of the presence of the GNA leader sequence. In the present study, the fusion protein, expressed under the control of the CaMV 35S promoter, also contained the GNA leader sequence that exports it to the phloem sap. A western blot-based quantification was necessary, as two bands react with anti-GNA antibody, Hv1a/GNA and a ∼10 kDa (similar to GNA) degradation product at an approximate proportion of 1:1. Therefore, results based on another commonly used method for protein quantification, ELISA, could be misleading in this case, as antibodies would recognize both, intact and degraded protein. Further improvements on protein stability would be necessary to prevent degradation following plant expression and ingestion by the aphid, enhancing its activity.

In contrast to *M. persicae*, *S. avenae* is a semi-specialist species and although it possesses proteolytic activity in the gut (Pyati et al., 2011), this aphid is not able to cleave the fusion protein as effectively. As a consequence, levels of Hv1a/GNA toxicity toward the grain aphid were higher than in *M. persicae* and also more evident, as GNA by itself did not affect its survival in artificial diet bioassays. It is therefore likely that expression of Hv1a/GNA in host plants of *S. avenae* would render them significantly more resistant to aphid infestation.

Recently, Bonning et al. (2014) fused the same spider venom peptide, Hv1a, to a luteovirid coat protein that is internalized by aphids following ingestion. The resulting fusion, CP-P-Hv1a, was toxic to four different homopteran species: *Acyrthosiphon pisum*, *Rhopalosiphum padi*, *Aphis glycines,* and *M. persicae*. These results indicate that Hv1a/GNA might also be effective against those other aphids, as contrary to the Hv1a peptide, the viral protein is innocuous to the insects. Compared to Hv1a/GNA, CP-P-Hv1a yielded apparently higher mortality to *M. persicae* when expressed in *Arabidopsis*, but with the drawback of not being effective against other major insect pests, such as *Heliothis virescens* larvae. This is because the viral coat protein is only likely to cross the gut barrier in insects that can act as vectors of luteoviruses, i.e., aphids. The outcome is that even though CP-P-Hv1a potentially poses lower risks of affecting non-target insect species, to which GNA can often be detrimental, it will also have a very limited spectrum of activity. On the other hand, Hv1a/GNA was previously shown to also be effective against the coleopterans *Tribolium castaneum* (Back, 2011) and *Leptinotarsa decemlineata*, and the lepidopteran *Mamestra brassicae* (Fitches et al., 2012), whilst presenting little hazard to honeybees (Nakasu et al., 2014). It is clear, however, that the levels of aphid control by Hv1a/GNA when expressed in transgenic plants are currently not sufficiently high to maintain aphid populations under economic thresholds.

Improvements in the fusion protein stability in the plant and after ingestion, coupled with increased expression in the phloem sap would potentially be beneficial for achieving this goal. Expressing Hv1a/GNA in suitable plant hosts for lepidopteran and coleopteran pests, e.g., *Heliothis virescens* and *Leptinotarsa decemlineata*, might further expand the range of insects that could be controlled by this biopesticide.

## Conflict of Interest Statement

The authors declare that the research was conducted in the absence of any commercial or financial relationships that could be construed as a potential conflict of interest.
