# Supplementary material for: Transgenic plants expressing ω-ACTX-Hv1a and snowdrop lectin (GNA) fusion protein show enhanced resistance to aphids
Source: Front Plant Sci. 2014 Nov 28;5:673. doi: 10.3389/fpls.2014.00673 (PMC4246675; doi:10.3389/fpls.2014.00673)
Supplement: Supplementary file 1 [file Data_Sheet_1.DOCX]

**Supplementary Material**

Hv1a/GNA sequence with codons optimized by ShineGene Molecular Biotech,Inc. for expression in *Arabidopsis thaliana*, inserted into plants.

ATGGCTAAGGCAAGTCTCCTCATTTTGGCCGCCATCTTCCTTGGTGTCATCACACCATCTTGCCTGAGTTCTCCTACTTGTATTCCTTCTGGACAACCTTGTCCTTATAATGAAAATTGTTGTTCTCAATCTTGTACTTTTAAGGAAAATGAAAATGGAAATACTGTTCAAAGATGTGATGCTGCTGCTGACAATATTTTGTACTCCGGTGAGACTCTCTCTACAGGGGAATTTCTCAACTACGGAAGTTTCGTTTTTATCATGCAAGAGGACTGCAATCTGGTCTTGTACGACGTGGACAAGCCAATCTGGGCAACAAACACAGGTGGTCTCTCCCGTAGCTGCTTCCTCAGCATGCAGACTGATGGGAACCTCGTGGTGTACAACCCATCGAACAAACCGATTTGGGCAAGCAACACTGGAGGCCAAAATGGGAATTACGTGTGCATCCTACAGAAGGATAGGAATGTTGTGATCTACGGAACTGATCGTTGGGCTACTGGAACTCACACCGGACTTGTTGGAATTCCCGCATCGCCACCCTCAGAGAAATATCCTACTGCTGGAAAGATAAAGCTTGTGACGGCAAAGTAA

Sequence translation: GNA precursor in black; ω-ACTX-Hv1a portion in red, the AAA linker in bold and GNA in blue.

MAKASLLILAAIFLGVITPSCLSSPTCIPSGQPCPYNENCCSQSCTFKENENGNTVQRCD**AAA**DNILYSGETLSTGEFLNYGSFVFIMQEDCNLVLYDVDKPIWATNTGGLSRSCFLSMQTDGNLVVYNPSNKPIWASNTGGQNGNYVCILQKDRNVVIYGTDRWATGTHTGLVGIPASP PSEKYPTAGKIKLVTAK
